# Dual-energy CT late arterial phase iodine maps for the diagnosis of acute non-occlusive mesenteric ischemia

**DOI:** 10.1007/s11547-024-01898-5

**Published:** 2024-10-15

**Authors:** Tommaso D’Angelo, Giuseppe M. Bucolo, Ibrahim Yel, Vitali Koch, Leon D. Gruenewald, Simon S. Martin, Leona S. Alizadeh, Thomas J. Vogl, Giorgio Ascenti, Ludovica R. M. Lanzafame, Silvio Mazziotti, Alfredo Blandino, Christian Booz

**Affiliations:** 1 Diagnostic and Interventional Radiology Unit, BIOMORF Department, University Hospital, Policlinico G. MartinoMessina, Via Consolare Valeria 1, 98100 Messina, Italy; 2 Department of Radiology and Nuclear Medicine, Erasmus MC, Doctor Molewaterplein 40, 3015 GD Rotterdam, The Netherlands; 3 Division of Experimental Imaging, Department of Diagnostic and Interventional Radiology, University Hospital Frankfurt, Theodor-Stern-Kai 7, 60590 Frankfurt Am Main, Germany

**Keywords:** Acute mesenteric ischemia; Dual energy; Computed tomography; Bowel diseases; Mesenteric vascular insufficiency

## Abstract

**Purpose:**

To evaluate the diagnostic accuracy of dual-energy CT (DECT) iodine maps in comparison to conventional CT series for the assessment of non-occlusive mesenteric ischemia (NOMI).

**Material and Methods:**

We evaluated data from 142 patients (72 men; 50.7%) who underwent DECT between 2018 and 2022, with surgically confirmed diagnosis of NOMI. One board-certified radiologist performed region of interest (ROI) measurements in bowel segments on late arterial (LA) and portal venous (PV) phase DECT iodine maps as well as LA conventional series, in both ischemic and non-ischemic bowel loops, using surgical reports as reference standard, and in a control group of 97 patients. Intra- and inter-reader agreement with a second board-certified radiologist was also evaluated. Receiver operating characteristic (ROC) curve analysis was performed to calculate the optimal threshold for discriminating ischemic from non-ischemic bowel segments. Subjective image rating of LA and PV iodine maps was performed.

**Results:**

DECT-based iodine concentration (IC) measurements showed significant differences in LA phase iodine maps between ischemic (median:0.72; IQR 0.52–0.91 mg/mL) and non-ischemic bowel loops (5.16; IQR 3.45–6.31 mg/ml) (*P* <.0001). IC quantification on LA phase revealed an AUC of 0.966 for the assessment of acute bowel ischemia, significantly higher compared to both IC quantification based on PV phase (0.951) and attenuation values evaluated on LA conventional CT series (0.828). Excellent intra-observer and strong inter-observer agreements were observed for the quantification of iodine concentration. Conversely, weak inter-observer agreement was noted for conventional HU assessments. The optimal LA phase-based IC threshold for assessing bowel ischemia was 1.34 mg/mL, yielding a sensitivity of 100% and specificity of 96.48%.

**Conclusion:**

Iodine maps based on LA phase significantly improve the diagnostic accuracy for the assessment of NOMI compared to conventional CT series and PV phase iodine maps.

## Introduction

Acute bowel ischemia (ABI) is a surgical emergency with a prevalence of about 0.1% of all acute admissions to the emergency department [1].

ABI mortality rate reaches 50–70% and has remained unchanged for the last decades, despite the advances in treatment [1].

The main etiopathogenetic classification is based on the presence or absence of vascular obstruction. Occlusive ischemia represents 60–80% and can be sustained by embolic or thrombotic events. On the other hand, non-occlusive mesenteric ischemia (NOMI) accounts for 20–30% of ABI. This type is mainly caused by systemic hypoperfusion syndromes (e.g., heart disease or systemic hypotensive shock), and its diagnosis can often be challenging [2].

Clinical presentation of ABI can be nonspecific and can mimic other causes of acute abdomen, potentially leading to delayed diagnosis. Nonetheless, early diagnosis and prompt intervention have been shown to improve patient outcome, accounting for a 58% reduction in mortality when intervention occurs within 12 h from the onset of symptoms [3].

CT represents the imaging modality of choice for the detection of ABI with a reported sensitivity ranging from 60 to 87% [4, 5]. Conversely, NOMI is more difficult to diagnose on conventional CT, and sensitivity can drop to 14.8% [6, 7].

With the advent of dual-energy CT (DECT), several studies have demonstrated improved tissue differentiation and characterization in the abdomen [8–17]. Colored iodine maps facilitate the assessment of tissue enhancement and vascularization compared to conventional single-energy CT value measurements, as they directly show contrast material enhancement without overlying background anatomical tissue density. DECT-based iodine mapping has shown to increase the perception of ABI-associated perfusion differences of bowel loops in an initial pilot study consisting of a small patient cohort [18]. However, in this study authors only assessed portal venous (PV) phase iodine maps. We hypothesized that late arterial (LA) phase iodine maps may allow for better discrimination of ischemic bowel from non-ischemic bowel segments and consequently for significantly higher diagnostic accuracy for the assessment of ABI compared to PV iodine maps and conventional CT. Thus, the purpose of this study was to evaluate the diagnostic accuracy of DECT LA phase iodine maps for the assessment of non-occlusive ABI compared to PV phase iodine maps and LA conventional CT series in a large patient cohort.

## Material and methods

This retrospective, monocentric study was approved by our institutional review board, and informed consent was obtained from all patients.

## Study population

We retrospectively reviewed our institutional database to identify consecutive patients who had undergone contrast-enhanced DECT scans of the abdomen due to high suspicion of ABI between January 2018 and June 2022. Administration of oral contrast material or deviation from intravenous contrast injection protocol led to patient exclusion. Patients with evidence of arterial occlusion or venous thrombosis were excluded.

**Fig. 1:**
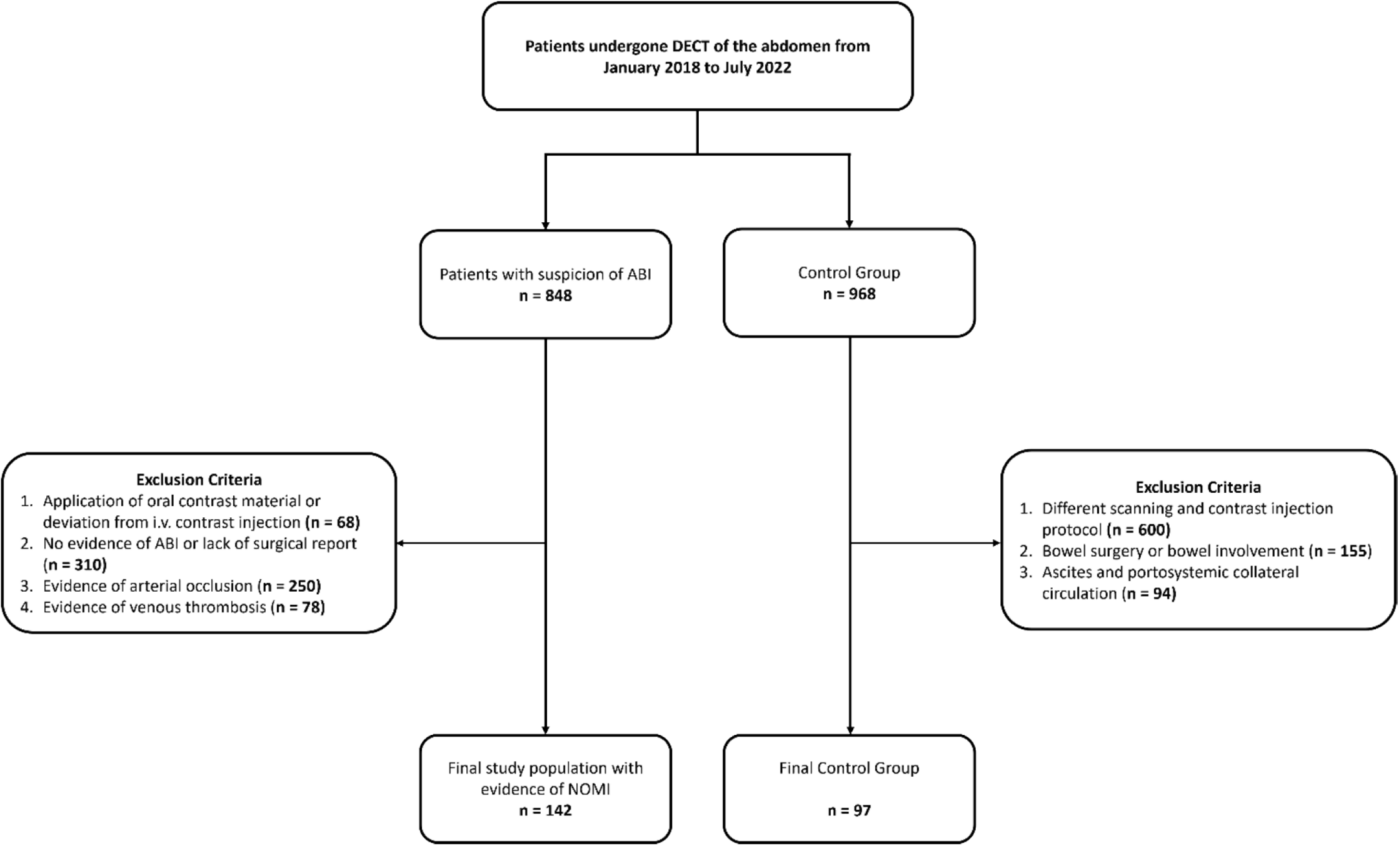
Flow chart overview of patient inclusion and exclusion criteria. *ABI* = *arterial bowel ischemia; DECT* = *dual-energy CT; NOMI* = *non-occlusive mesenteric ischemia*

Only patients with surgical procedural reports confirming the diagnosis of NOMI were included in the final patient cohort. Figure 1 displays the selection process of this study.

A control group of patients who had undergone contrast-enhanced DECT scans in the same period was identified in our PACS and included.

## DECT scan protocol

All included abdominal DECT examinations were performed on a third-generation dual-source DECT scanner (SOMATOM Force, Siemens Healthineers, Forchheim, Germany). Patients were examined in the supine position, and anteroposterior scout radiographs were obtained ranging from the diaphragm to the pubic symphysis. Image acquisition was performed in craniocaudal direction during inspiratory breath-hold.

The study protocol consisted of unenhanced, LA, and PV contrast-enhanced phase scans. Contrast media (Iomeron 400 mgI/ml, Bracco, Milan, Italy) was intravenously injected at a dose of 1.3 mL per kilogram body weight and at a flow rate of 3 mL/s, through a superficial vein of the forearm. No oral contrast material was administered.

A bolus tracking ROI was positioned on the descending aorta and the LA phase started 15 s after reaching of the 100 HU trigger threshold.

PV phase scans were subsequently acquired at a fixed time delay of 75 s. The following settings were used for dual-source DECT imaging: tube A, 90 kV and 190 mAs per rotation; tube B, Sn 150 kV with tin filter and 95 mAs per rotation; rotation time, 0.5 s; pitch, 0.6; and collimation, 2 × 192 × 0.6 mm.

## Image reconstruction and iodine map generation

All CT image series were reconstructed using a medium-soft reconstruction kernel (Qr40f) with 3.0-mm section collimation and 2.0-mm increment in the axial and coronal planes. The scanner automatically generated linearly blended images with a weighted factor of 0.6 (M_0.6) between the 90-kV and 150-kV image datasets, to simulate conventional 120-kVp images in both the axial and coronal planes.

DECT material decomposition image series of the LA and PV acquisitions were post-processed on a DECT workstation using vendor default settings (syngo.via, version VB10B, Siemens Healthineers) to generate iodine maps in both the axial and coronal planes with 3.0-mm section collimation and 2.0-mm increment.

## Quantitative image evaluation

One board-certified radiologist (*BLINDED* with 7 years of experience in abdominal imaging) evaluated bowel loops by manually placing ten 5 mm^2^ regions of interest (ROI) in the bowel wall of surgically proven ischemic segments and ten ROI in the bowel wall of non-ischemic segments based on the surgical reports and after close consultation with the corresponding surgeons. This process began with a focused assessment of the abdominal quadrant, ensuring a precise evaluation of the affected area. In particular, an ischemic loop was identified by comparing CT signs of reduced enhancement with surgeon’s report and recommendations.

This was simultaneously performed on axial iodine maps based on LA and PV phases to measure iodine concentration (IC) in mg/mL and on axial conventional CT images of the LA phase to generate attenuation numbers (HU).

**Fig. 2:**
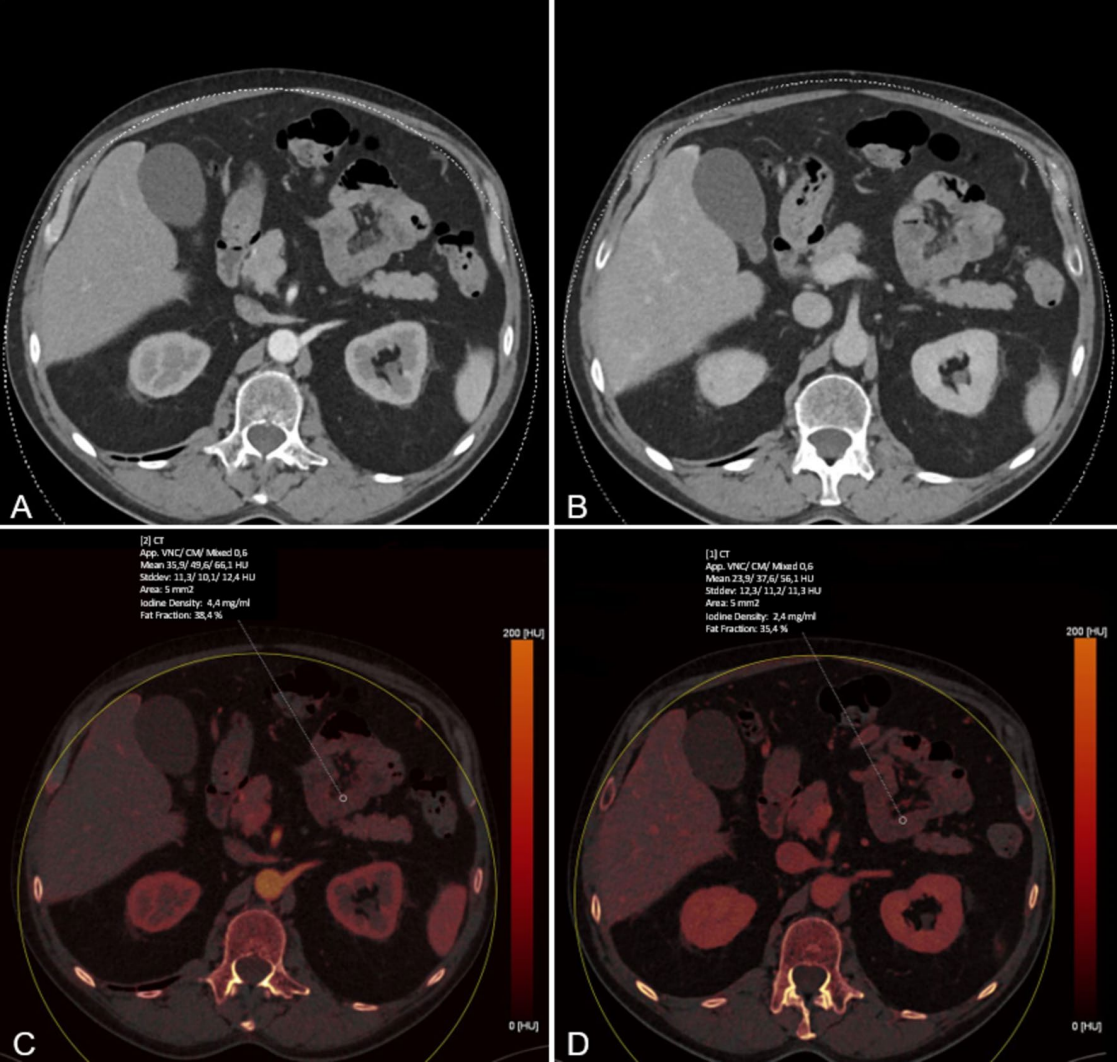
Conventional linearly blended late arterial (LA) and portal venous (PV) phase dual-energy CT scans (**A** and **B**, respectively) LA and PV iodine maps (**C** and **D**, respectively) were created using dedicated postprocessing software. Iodine concentration measurements in both phases and Hounsfield unit (HU) measurements were obtained by placing ten regions of interest (ROI, 5 mm^2^) in surgically proven ischemic and ten ROI in surgically proven non-ischemic bowel segments, respectively. Values were automatically computed by the postprocessing software.

Figure 2 provides a sample ROI measurement that shows IC assessment on both LA and PV phases. Mean values of measurements were used for the statistical analysis.

In the control group, an identical procedure for placing ROIs was performed on the walls of arbitrarily selected bowel loops.

Intra- and inter-reader agreement were assessed in 40 randomly selected patients, split evenly between the ischemic (n. 20) and control (n. 20) groups. Inter-reader agreement was performed independently by a second board-certified radiologist with 8 years of experience in abdominal imaging (*BLINDED*).

## Subjective image evaluation

Image evaluation was performed with a conventional picture archiving and communication system workstation (Centricity, version 4.2; GE Healthcare, Solingen, Germany). Five board-certified radiologists (*BLINDED* with 6–10 years of experience in abdominal imaging) evaluated LA and PV phase iodine maps. Criteria were the suitability of images for ABI assessment in general, the image contrast, and the image noise. Each reader was free to adjust window settings and scroll through the whole stack of CT series. Five-point Likert scales (1, unacceptable; 5, excellent) were used for each criterion.

## Statistical analysis

The normality of data distribution was assessed using the Kolmogorov–Smirnov test. All continuous values showed non-normal distribution and were reported as medians with interquartile ranges (IQR).

The Wilcoxon matched-pairs test was used for non-normally distributed variables. A statistically significant difference was indicated by a *P* value less than 0.05. All statistical analyses were performed on a commercially available software (MedCalc 18.2, MedCalc Software Ltd, Ostend, Belgium). Bland–Altman plots together with intraclass correlation coefficient (ICC) were used to assess intra- and inter-reader agreement for quantitative image evaluation. Interpretation of ICC results followed this scale: ICC < 0.40 = poor correlation, ICC 0.41–0.59 = fair correlation, ICC 0.60–0.74 = good correlation, and ICC > 0.75 = excellent correlation [19].

Receiving operating characteristic (ROC) curve analysis was used to establish the threshold value of IC to distinguish between ischemic and non-ischemic bowel loops both for LA and PV phases and for attenuation values (HU) obtained in the conventional LA phase [20]. The method of Delong et al. was used to compare the area under the curves (AUCs) among the IC analysis in LA and PV phases and the HU values in the conventional series [21]. Accuracy, sensitivity, and specificity values with corresponding 95% confidence intervals (CI) were determined by Youden’s J statistics. For subjective image evaluation, inter-reader agreement was analyzed by calculation of weighted Fleiss’ κ according to Landis and Koch [22].

## Results

### Population characteristics

We evaluated 848 patients who underwent DECT of the abdomen in the suspicion of ABI. Administration of oral contrast material or deviation from intravenous contrast injection protocol led to the exclusion of 68 patients. Other 310 patients were excluded because of no evidence of ischemia. The other exclusion criteria were evidence of arterial occlusion (250 patients) and venous thrombosis (78 patients).

Our final study cohort consisted of 142 patients [median age: 69 (IQR 58 – 81); age range: 38–92 years; 72 men (50.7%)], with surgical evidence of NOMI, who were therefore considered true positive (Table 1).

**Table 1:** Patient demographics

Characteristic	Patients with NOMI (*n* = 142)	Control group (*n* = 97)
*Sex, no*. (%)		
Male	72 (51%)	54 (55.6%)
Female	70 (49%)	43 (44.4%)
Age; IQR (y)	69; IQR 58–81	71; IQR 65.75–76
Male	71.50; IQR 62.0–82.5	71; IQR 66—76
Female	66; IQR 55–79	72; IQR 62.25–79.75
BMI; IQR (kg/m^2^)	25.89; IQR 23.55–27.48	25.53; IQR 22.98–26.87
Male	26.18; IQR 23.09–28.50	26.56; IQR 24.49–27.40
Female	25.83; IQR 23.77–27.15	23.51; IQR 21.16–25.51

*Values are expressed as medians and interquartile ranges (IQR). BMI: body mass index*

For the control group, we reviewed 968 patients who had undergone abdomen DECT scans for other diseases. Different scanning parameters or contrast injection protocol led to exclusion of 600 patients. One hundred and fifty-five (155) patients were excluded due to bowel surgery or other bowel involvement. Ninety-four patients were excluded because of evidence of ascites and portosystemic collateral circulation. The final control group consisted of 97 patients [median age: 71 (IQR 65.75—76) years; age range: 46–95 years; 54 men (55.6%)], who had undergone imaging due to the staging or follow-up of hepatocellular carcinoma (HCC) (*n* = 34), renal cell carcinoma (RCC) (*n* = 22), pancreatic adenocarcinoma (*n* = 14), melanoma (*n* = 13), cholangiocarcinoma (*n* = 9), and characterization of unknown liver nodules (*n* = 5).

## Quantitative image evaluation

Quantitative assessment of bowel wall enhancement showed excellent intra-observer agreement. Inter-observer agreement showed excellent results for the quantification of iodine concentration, while the quantitative HU assessment of the bowel wall showed a significant mean difference (Table 2**)**.

**Table 2:** Intra- and inter-observer agreement analysis of the measurements performed on 20 patients of the NOMI group and 20 patients of the control group

	Intra-observer			Inter-observer		
	Mean error (LoA)	ICC	*p* value	Mean error (LoA)	ICC	*p* value
*IC LA*						
NOMI Group	− 0.09(-0.74, 0.45)	0.8694	0.0898	− 0.05(-0.49, 0.38)	0.7833	0.2867
Control Group	0.11(− 0.73, 1.27)	0.8202	0.1739	0.05(− 0.66, 0.75)	0.9821	0.5817
*IC PV*						
NOMI Group	− 0.08(-0.58, 0.79)	0.8977	0.0897	0.15(− 0.68, 0.97)	0.7788	0.1387
Control Group	0.15(− 0.91, 1.17)	0.9185	0.0759	0.15(− 0.91, 1.22)	0.8759	0.2255
*HU LA*						
NOMI Group	− 10.25(-25.77, 17.22)	0.8030	0.0613	− 8.10(− 25.55, 9.35)	0.8161	0.0007*
Control Group	15.36(− 10.37, 31.71)	0.7801	0.0508	− 7.91(− 31.96, 16.14)	0.6182	0.0095*

*HU: Hounsfield unit; IC: iodine concentration; ICC: intraclass correlation coefficient; LA: late arterial; LoA: Limits of agreement. PV: portal venous. Asterisk indicates statistical significance*

Significant differences between ischemic and non-ischemic intestinal segments were seen in all datasets.

In LA phase iodine map reconstructions, the median ischemic bowel wall iodine concentration (BWIC) was 0.72 mg/ml (IQR 0.52–0.91) compared to 5.16 mg/ml (IQR 3.45–6.31) in non-ischemic segments (*P* < 0.0001), corresponding to IC reduction of 86%.

Median BWIC in the PV phase was also significantly reduced (57%) in ischemic bowel loops (1.12 mg/ml; IQR 0.92–1.38), compared to non-ischemic segments (2.61 mg/ml; IQR 1.92–3.45; *P <* 0.0001).

Similarly, attenuation values in the conventional LA phase significantly differed between ischemic (39.75 HU; IQR: 29.60–51.30) and non-ischemic bowel segments (55.85 HU; IQR: 47.10–64.10; *P* < 0.0001), with a reduction of about 29%.

Furthermore, the comparison of BWIC between the ischemic and the control group demonstrated a significant difference. Median BWIC showed a significant reduction in LA phase (0.72 mg/ml; IQR 0.52–0.91 vs 4.98 mg/ml; IQR 3.18–6.92 of control group) and in PV phase (median IC 1.12 mg/ml; IQR 0.92–1.38 vs 2.68 mg/ml; IQR 2–3.46 of control group) (*P* < 0.0001). Similarly, attenuation values in LA phase were significantly reduced in ischemic segments (median 39.75 HU; IQR 29.60–51.30) compared to the control group segments (median 57.2 HU; IQR 48.45–64.20) (*P* < 0.0001).

**Fig. 3:**
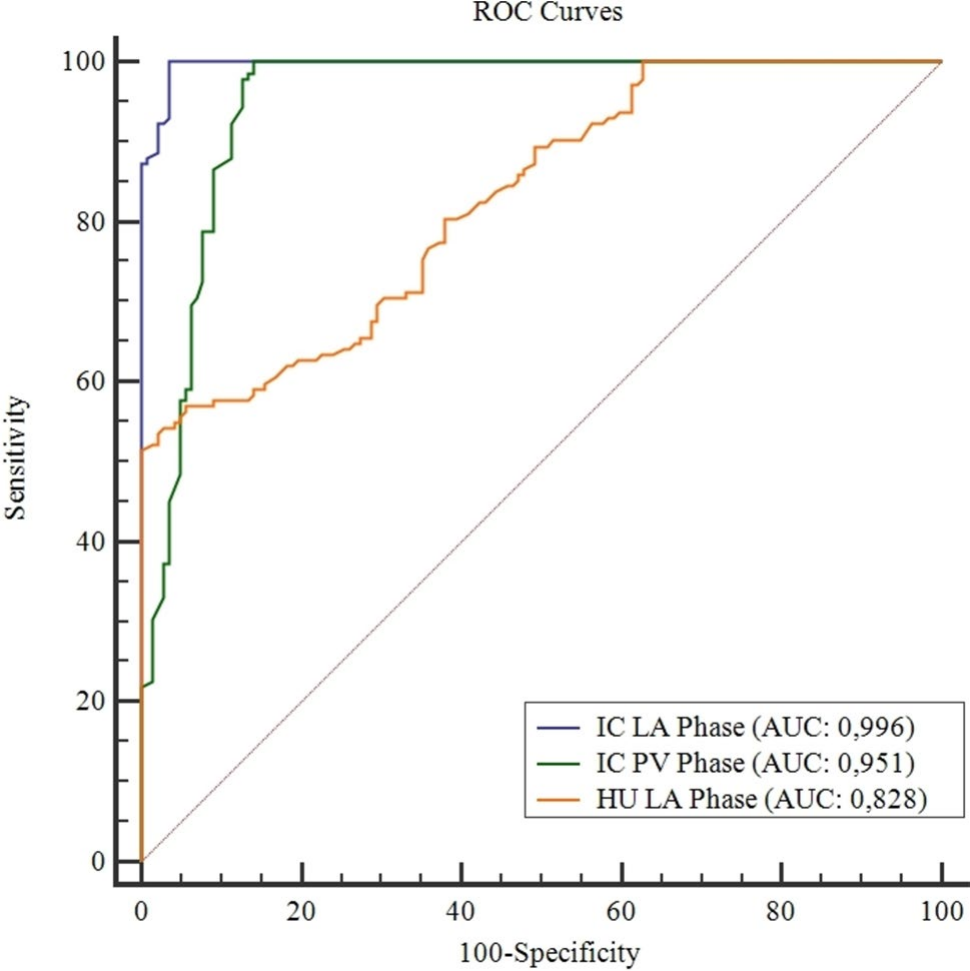
The comparison of receiver operating characteristic (ROC) curves shows the diagnostic performance of iodine concentration (IC) measurements in late arterial (LA) phase and in portal venous (PV) phase together with Hounsfield unit (HU) assessment in LA phase. The numerical values show the accuracy of each method

The highest diagnostic accuracy for the detection of ischemic bowel loops was obtained by LA phase iodine maps (AUC: 0.996) (Fig. 3). BWIC measurements in the PV phase (AUC: 0.951) and conventional attenuation measurements (AUC 0.828) presented significantly lower values (*P* < 0.0003 and *P* < 0.0001, respectively).

**Fig. 4:**
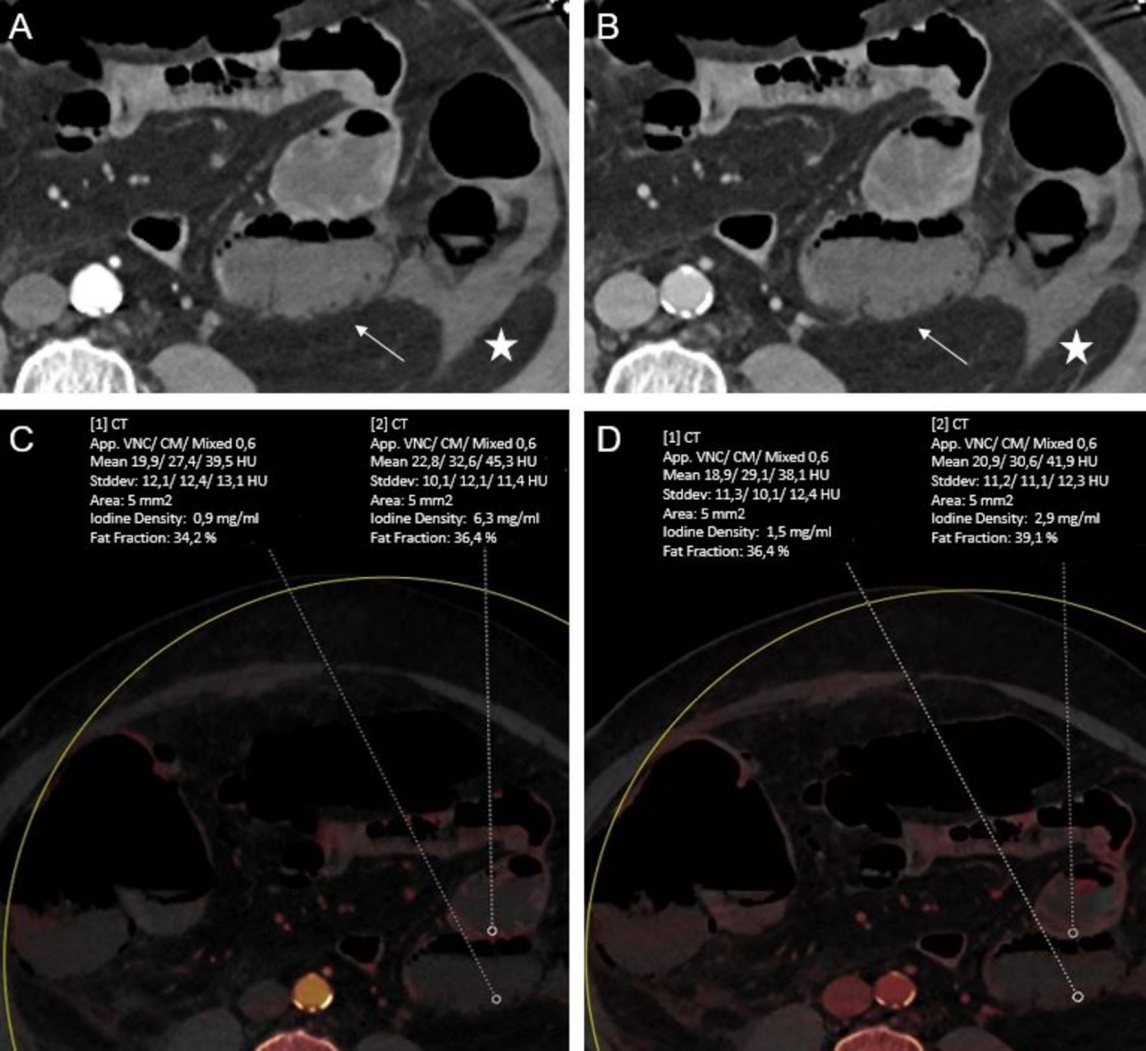
60-year-old woman with acute abdomen and elevated lactate. Conventional transversal late arterial (LA, **A**) and portal venous (PV, **B**) demonstrate free intraabdominal fluid (*star*) next to a small bowel loop (*arrow*). Corresponding dual-energy CT iodine maps based on LA and PV phase (**C** and **D**, respectively) demonstrate a greater difference in iodine concentration (IC) reduction of this small bowel loop compared with adjacent segments in the LA phase (LA: 0.9 vs 6.3 mg/ml; PV: 1.5 vs 2.9 mg/ml), indicating significant ischemia which was proven by surgery. CT value measurements showed only a slight reduction (LA: 39.5 vs 45.3; PV: 38.1 vs 41.9)

**Fig. 5:**
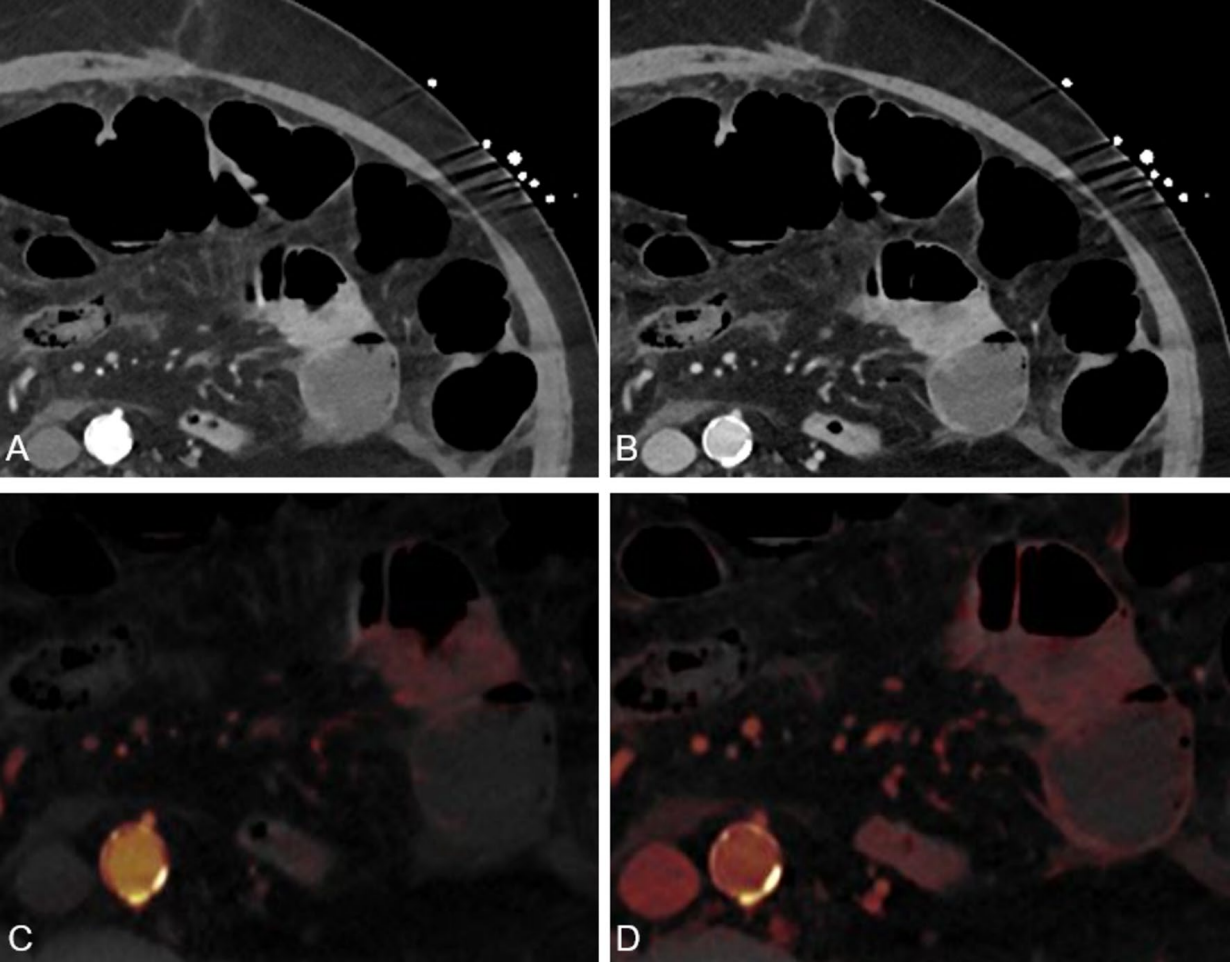
67-year-old man suffering from acute abdomen and left-sided abdominal pain. Transversal conventional late arterial (LA) phase (**A**) and portal venous (PV) phase. (**B**) CT series show slightly decreased enhancement of a small bowel loop in the left abdomen in a surgically proven case, particularly in the LA phase. At the visual analysis, LA phase iodine maps (**C**) were significantly better rated regarding bowel ischemia conspicuity assessment, contrast and image noise compared to PV phase iodine maps (**D**) (all categories, *P* < .001). While LA phase iodine maps show a distinct visual difference between ischemic and non-ischemic bowel, there is only a slight visual difference on PV phase iodine map, which makes the diagnosis challenging

On LA phase, the threshold BWIC of 1.34 mg/dl yielded sensitivity of 100% (95% CI, 97.40–100) and specificity of 96.48% (95% CI, 92–98.80) for differentiation between ischemic and non-ischemic bowel loops (Figs. 4 and 5).

On PV phase, the threshold BWIC of 1.57 mg/dl showed a sensitivity of 100% (95% CI, 97.40–100) and a specificity of 85.92% (95% CI, 79.10–91.20).

On LA phase conventional series, the threshold attenuation of 39.90 HU (Youden index J: 0.514) yielded a sensitivity of 51.41% (95% CI, 42.90–59.90) and a specificity of 100% (95% CI, 97.40–100).

Results of the diagnostic performance analysis are summarized in Table 3.

**Table 3:** Quantitative Image analysis results.

	IC LA phase	IC PV phase	HU LA phase
Ischemic bowel	**0.72**	**1.12**	**39.75**
	IQR 0.52–0.91 mg/ml	IQR 0.92–1.38 mg/ml	IQR: 29.60–51.30 HU
Non-ischemic bowel	**5.16**	**2.61**	**55.85**
	IQR 3.45–6.31 mg/ml	IQR 1.92–3.45 mg/ml	IQR: 47.10–64.10 HU
Control group	**4.98**	**2.68**	**57.2**
	IQR 3.18–6.92 mg/ml	IQR 2–3.46 mg/ml	IQR 48.45–64.20 mg/ml
Optimal thresholds	1.34*	1.57*	39.9*
	(Youden J: 0.965)	(Youden J: 0.859)	(Youden J: 0.514)
Sensitivity (%)	100 (97.40—100)	100 (97.40—100)	51.41 (42.90—59.90)
Specificity (%)	96.48 (92—98.80)	85.92 (79.10 – 91.20)	100 (97.40—100)
Accuracy (%)	99.6	95.1	82.8

Median values are in bold. Optimal thresholds for differentiating ischemic VS. non-ischemic bowel segments are indicated by asterisk. Numbers in parentheses are 95% CI.

*IQR: interquartile range; IC: iodine concentration; LA: late arterial; PV: portal venous; HU: Hounsfield unit*

## Subjective image evaluation

**Fig. 6:**
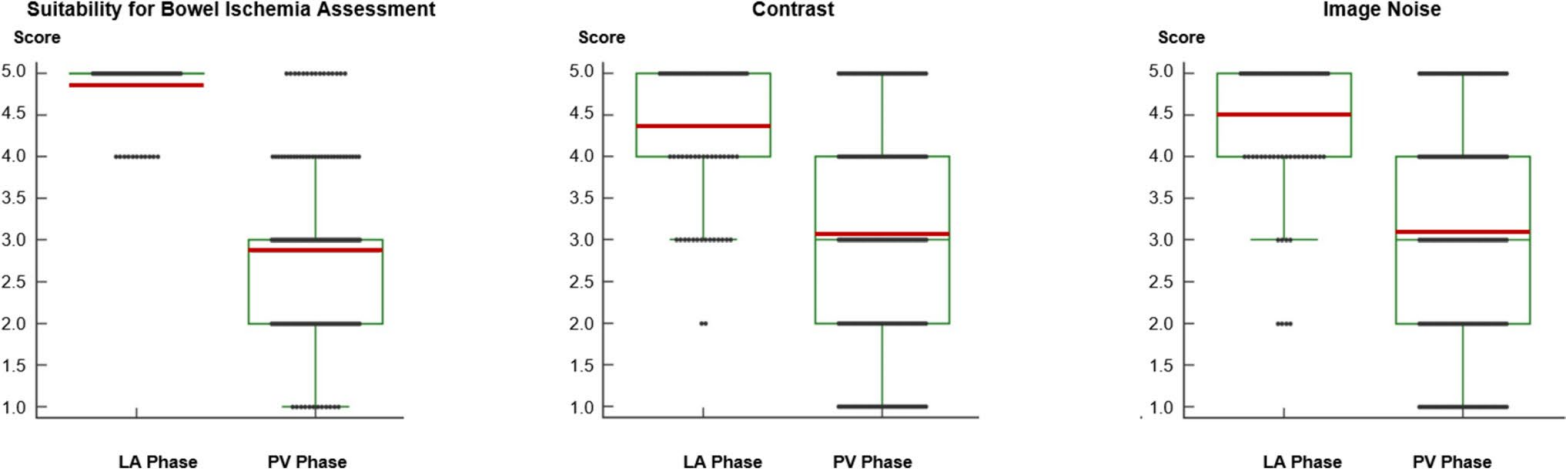
Box and whisker plots show subjective image ratings about the general suitability for bowel ischemia conspicuity assessment, contrast and image noise of late arterial (LA) and portal venous (PV) phase iodine maps. Median scores are displayed as horizontal red lines. LA phase iodine maps were rated better than PV phase iodine maps with higher mean scores for all rating categories (all *P* < .001)

Subjective image rating demonstrated significantly better suitability of LA phase iodine maps compared to PV phase iodine maps for the assessment of ABI [mean scores of 4.90 vs. 2.95 (*P* < 0.001)] (Fig. 6).

Contrast enhancement in LA and PV phase iodine maps was rated with mean scores of 4.38 vs. 3.12, respectively (*P* < 0.001). Image noise had mean scores of 4.55 vs. 3.17, respectively, for LA phase and PV phase iodine maps (*P* < 0.001).

Inter-reader agreement was excellent for all parameters in both LA phase (all κ > 0.89; 95% CI, 0.84–0.96) and PV phase iodine maps (all κ > 0.82; 95% CI, 0.75–0.90).

## Discussion

We demonstrated that BWIC measurements based on LA phase DECT scanning provide significantly higher diagnostic accuracy for the assessment of ABI compared to BWIC performed on PV phase or to conventional attenuation values. In addition, LA phase iodine maps received superior ratings regarding suitability of images for subjective evaluation of ABI compared to PV phase iodine maps, further emphasizing their potentially important role for the clinical use.

ABI clinical presentation is variable and early diagnosis is critical since delayed treatment may lead to worsening of patient outcome and higher mortality rate [5]. In particular, different studies have shown that early treatment (i.e., endovascular or surgical), performed within the first 12 h following the onset of AMI symptoms, has a significantly reduced mortality rate compared to those who underwent delayed surgical intervention (14% vs. 75%) (3).

CT angiography represents the current imaging method of choice for diagnosis of ABI, and it should be timely performed in patients presenting clinical symptoms [23]. Early CT findings in ABI consist of various morphologic changes such as reduction of visceral enhancement, mucosal hemorrhage, intestinal dilatation, and thickening of the bowel wall associated with perifocal ascites [7]. On the other hand, in terminal stage typical findings are mesenteric stranding, vascular engorgement, pneumatosis, portal venous gas, and free abdominal air [24].

While diagnosis of superior mesenteric artery occlusion or portal vein thrombosis causing ABI can be readily assessed, radiological diagnosis of ABI can often be particularly challenging in early-stage NOMI, due to a substantial overlap with early findings of differential diagnoses such as inflammatory and infectious bowel diseases [6, 25]. For this reason, reduced visceral enhancement is still considered the most specific findings for the diagnosis of NOMI.

With the advent of DECT, several studies have demonstrated improved tissue differentiation and lesion detection in a number of abdominal conditions [8–17]. Color-coded iodine maps may also facilitate the assessment of bowel wall vascularization compared to conventional single-energy CT attenuation measurements, since they show directly contrast material distribution without any superimposed anatomical structures that contribute to attenuation values.

A recent animal model study showed that DECT can significantly improve the conspicuity of the ischemic bowel loops compared with conventional CT [26].

Similarly, Lourenco et al. demonstrated higher sensitivity of DECT iodine maps for the assessment of ABI in comparison with conventional CT, with a reduction of false positive diagnosis [18]. However, in their study, the authors assessed IC measurements on PV phase scan only, and in a small cohort of true positive patients, in whose only seven out of sixteen total patients had a definitive diagnosis of NOMI.

Moreover, the iodinated contrast dose was not adjusted for the patient body weight, which may result in less homogenous and reproducible patterns of intestinal mural enhancement.

Our study cohort included 142 patients with definite diagnosis of NOMI who underwent DECT on both LA and PV phases.

Compared to the results from Lourenco et al., we found concordant mean BWIC on the PV phase and CT attenuation values on the LA phase, for both ischemic and non-ischemic bowel segments. However, our results show a better diagnostic accuracy when BWIC measurement is performed on LA phase iodine maps, which represents new knowledge compared to previous studies. In addition, we firstly identified optimal cutoff values for differentiation of ischemic and non-ischemic bowel segments. In this setting, a cutoff BWIC value of 1.34 mg/dl based on LA phase showed sensitivity and specificity of 100% and 96.50%, respectively, potentially representing an accurate method for the diagnosis of NOMI.

Patients may benefit from this diagnostic performance especially in case of early-stage NOMI, when clinical outcome may be improved due to earlier detection of reduced bowel wall perfusion, with subsequent accelerated therapy initiation. Moreover, this particular DECT algorithm may be most valuable in cases where intramural hemorrhage potentially mimics mural thickening and enhancement, or to help differentiate effective from ineffective reperfusion, which is a phenomenon often occurring in patients with NOMI [27, 28].

We believe that DECT LA phase iodine map and the corresponding BWIC measurements should be performed in the suspicion of ABI, especially when superior mesenteric artery occlusion or portal venous thrombosis is absent.

However, there are limitations in our study that need to be addressed. Firstly, all CT scans were performed on a third-generation dual-source DECT scanner. Because improved technical and computational material differentiation, soft-tissue contrast, and spatial resolution have improved significantly, results from former dual-source platforms or other DECT scanners may show different diagnostic accuracy and should be evaluated in further studies. Secondly, our study design was retrospective, which may have influenced the results. Thirdly, the ROI placement can be difficult in some cases due to the flexible anatomy of bowel segments, which may affect the measurement validity. In order to minimize this error, the ROI placement was performed by experienced radiologists, who were aware of the surgical report. Fourthly, BWIC remains dependent on patient-related factors, even when standardized contrast-medium protocols are adopted, as in this study. Particularly, in the case of severely obese patients, the peak arterial parenchyma enhancement could be affected by the injection duration, while the venous enhancement depends solely on the amount of iodine injected [29]. Additionally, the results of this study are only valid for the specific scan protocol applied; different diagnostic performances may result from using alternative scan or contrast injections protocols. Furthermore, reperfusion phenomena and intramural hemorrhage may have altered the mucosal enhancement, thus modifying BWIC values [28, 30]. Finally, in our study, we did not distinguish between normally collapsed and distended loops. The former may exhibit higher values compared to the latter [31]. Further research on this topic could benefit from making this distinction to enhance understanding and accuracy.

In conclusion, DECT iodine maps based on LA phase significantly improve the diagnostic accuracy for the diagnosis of NOMI compared to conventional CT series and PV phase iodine maps. Therefore, we believe that LA phase iodine map reconstruction should be performed in case of suspected ABI.

## Data Availability

The full dataset will not be provided for publication at this moment due to further upcoming research projects related to department of University Hospital Frankfurt.

## Abbreviations

ABI: Acute bowel ischemia
AUC: Area under the curve
BWIC: Bowel wall iodine concentration
CI: Confidence interval
DECT: Dual-energy CT
HU: Hounsfield unit
IC: Iodine concentration
LA: Late arterial
NOMI: Non-occlusive mesenteric ischemia
PV: Portal venous
ROC: Receiving operating characteristic
ROI: Region of interest
VMI: Virtual monoenergetic imaging
